# Testosterone plus lifestyle therapy improves skeletal muscle glycolysis in older men with obesity and hypogonadism

**DOI:** 10.3389/fendo.2025.1719749

**Published:** 2026-02-09

**Authors:** Viola Viola, Marlene Aguilar, Maria Liza Duremdes Nava, Alessandra Celli, Reina Armamento-Villareal, Yoann Barnouin, Nicola Napoli, Nagireeddy Putluri, Clifford Qualls, Dennis T. Villareal

**Affiliations:** 1 Center for Translational Research on Inflammatory Diseases, Michael E DeBakey VA Medical Center, Houston, TX, United States; 2 Division of Endocrinology, Diabetes and Metabolism, Baylor College of Medicine, Houston, TX, United States; 3 Operative Research Unit of Osteo-Metabolic and Thyroid Diseases, Fondazione Policlinico Universitario Campus Bio-Medico, Rome, Italy; 4 Division of Bone and Mineral Diseases, Washington University in St Louis, St. Louis, MO, United States; 5 Department of Molecular and Cellular Biology, Baylor College of Medicine, Houston, TX, United States; 6 Advanced Technology Core, Baylor College of Medicine, Houston, TX, United States; 7 Department of Mathematics and Statistics, University of New Mexico, Albuquerque, NM, United States

**Keywords:** glycolysis, hypogonadism, lifestyle, muscle metabolism, obesity, testosterone

## Abstract

**Objective:**

Weight loss in older men with obesity and hypogonadism accelerates musculoskeletal decline, yet the underlying metabolic mechanisms remain unclear. Testosterone replacement therapy (TRT), when added to lifestyle therapy (LT), mitigates this decline, but its metabolic basis has not been defined. We examined skeletal muscle metabolomic adaptations to LT with or without TRT, focusing on glycolysis, the pentose phosphate pathway (PPP), the tricarboxylic acid (TCA) cycle, and carnitine metabolism to identify dominant pathways of metabolic adaptation.

**Design:**

Randomized, double-blind, placebo-controlled trial (LITROS).

**Methods:**

Eighty-three men aged 65 years or older with obesity (BMI ≥30 kg/m^2^), hypogonadism (testosterone <10.4 nmol/L), and frailty (Physical Performance Test score ≤31) were randomized to 26 weeks of LT plus TRT (LT+TRT) or LT plus placebo (LT+Pbo). A metabolomic substudy was performed in 44 participants, who underwent serial biopsies of the vastus lateralis for targeted LC-MS/MS analysis of intermediates in glycolysis, PPP, TCA cycle, and carnitine metabolism.

**Results:**

Among the pathways examined, only glycolysis showed a consistent and significant response to LT+TRT versus LT+Pbo (between-group *p* = 0.005). This response was characterized by increases in preparatory (G6P/F6P, FBP) and payoff (3PG, 2PG, PEP) intermediates, along with higher lactate concentrations, whereas pyruvate remained stable. The PPP showed limited changes, and neither the TCA cycle nor carnitine metabolites exhibited consistent patterns. In LT+TRT, the glycolysis factor score was positively correlated with VO_2_peak (r=0.47, *p* = 0.04) and inversely correlated with triglycerides (r=–0.52, *P* = 0.01) and the metabolic syndrome score (r=–0.48, *p* = 0.02). No significant correlations were observed in LT+Pbo.

**Conclusions:**

TRT during LT selectively enhances skeletal muscle glycolysis, identifying glycolic activation as the dominant metabolic adaptation in this mechanistic study. By increasing glycolytic flux under calorie restriction, TRT may produce efficient ATP generation while conserving amino acids, supporting muscle and bone preservation and improving aerobic and cardiometabolic function in older men with obesity and hypogonadism.

## Introduction

Obesity is a major global health issue that affects millions of adults, with particularly high prevalence in older populations (1, 2). As obesity continues to rise in older adults, it contributes to an escalating burden of comorbidities such as frailty, sarcopenia, and osteoporosis (3–5), all of which adversely impact quality of life and increase healthcare costs. These age-related declines in physical function are further compounded by reduced levels of anabolic hormones, particularly testosterone (6). In healthy aging men, total testosterone levels generally fall toward the lower end of the adult reference range (approximately 12–35 nmol/L), whereas levels frequently fall below 10.4 nmol/L in obesity-related functional hypogonadism (7–10) Testosterone is critical for maintaining muscle mass, strength, and bone mineral density (BMD) in men (11, 12), and it also regulates metabolic pathways that support energy homeostasis—including glycolysis, the pentose pathway, and carnitine-dependent fatty-acid transport (13–16). When testosterone levels decline with age and are further suppressed by obesity, these metabolic processes become dysregulated, contributing to metabolic inflexibility and vulnerability to muscle and bone loss Metabolomic studies have demonstrated that low testosterone impairs glycolysis, the pentose phosphate pathway (PPP), and carnitine metabolism (14, 15, 17), highlighting the need for interventions that restore both anabolic signaling and metabolic resistance in older men with obesity.

Lifestyle therapy (LT), which includes calorie restriction (CR) and physical activity (PA), remains the foundational strategy for managing obesity, producing weight loss and improvements in physical and cardiometabolic function (18–21). However, LT alone may be insufficient to preserve muscle mass and strength in older adults with obesity, particularly in the presence of hypogonadism. Low testosterone blunts the anabolic signaling required to maintain skeletal muscle and bone (22), making testosterone deficiency an important contributor to impaired musculoskeletal health in this population. Combining testosterone replacement therapy (TRT) with LT offers a promising approach to counteract the catabolic effects of obesity and aging.

The Lifestyle Intervention and Testosterone Replacement in Obese Seniors (LITROS) trial (NCT02367105) was designed to test whether TRT augments the effects of LT on physical function in older men with obesity and hypogonadism. In the parent trial, scores on the Physical Performance Test (PPT) improved similarly between LT plus TRT and LT plus placebo, whereas peak oxygen consumption (VO_2_peak) increased more with TRT (23). TRT also helped preserve lean mass and hip BMD during weight reduction (23). Subsequent analyses reported no additional benefits for cardiometabolic outcomes (24), while transcriptomic profiling demonstrated that TRT prevented LT-induced downregulation of genes critical for muscle and bone anabolism and blunted LT-associated downregulation of skeletal muscle metabolic pathways, suggesting broader preservation of metabolic function (22).

These findings provided the rationale for the present muscle metabolomics substudy. We investigated whether adding TRT to LT modifies the skeletal-muscle metabolome, focusing on pathways that regulate energy production and substrate utilization. Specifically, we profiled glycolysis, the PPP, carnitine metabolism, and the tricarboxylic acid (TCA) cycle to determine whether pathway-level adaptations differed between LT with TRT and LT with placebo in older men with obesity and hypogonadism.

## Methods

### Study design and participants

This report is a predefined substudy of the LITROS trial (ClinicalTrials.gov, NCT02367105), which focused on skeletal muscle metabolomics, with selected clinical outcomes summarized to characterize the substudy cohort. The design and outcomes of the parent trial have been described in detail previously (23).

**Figure 1:**
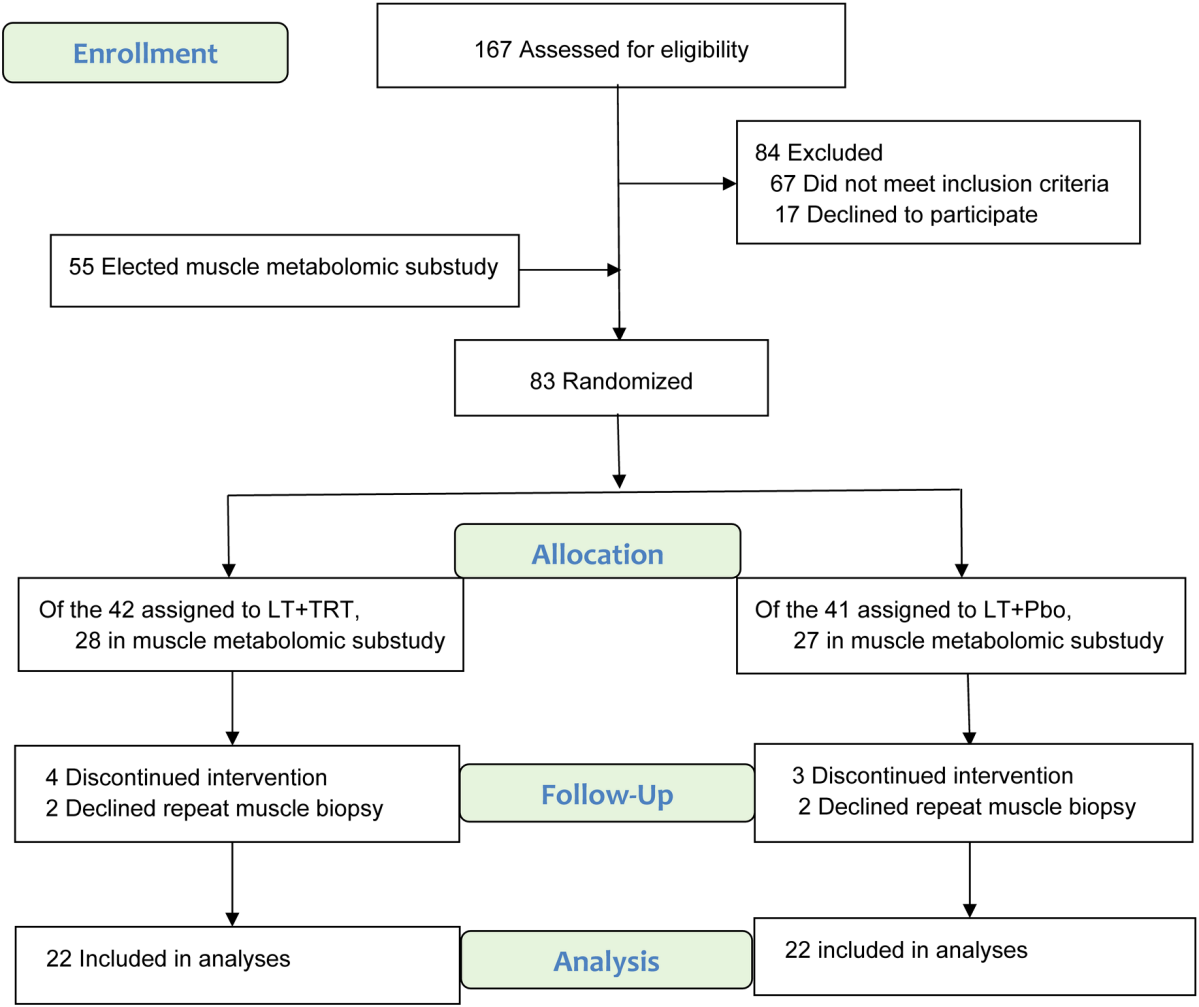
Screening, Randomization, and Follow-up. Groups: LT+TRT (lifestyle therapy + testosterone replacement therapy) and LT+Pbo (lifestyle therapy + placebo).

Of the 83 men randomized in the parent trial, 55 consented to participate in the metabolomics substudy and underwent muscle biopsy (**Figure 1**). Twenty-eight were assigned to lifestyle therapy plus testosterone replacement therapy (LT+TRT) and 27 to lifestyle therapy plus placebo (LT+Pbo). After accounting for discontinuations and participants who declined repeat biopsy, 22 in each group provided paired baseline and 6-month muscle samples suitable for metabolomics analyses.

The study was conducted at the Michael E. DeBakey Veterans Affairs Medical Center (MEDVAMC) in Houston, Texas, with approval from the Institutional Review Board of Baylor College of Medicine and the MEDVAMC Research and Development Committee (IRB protocol number H-35267). Participants were identified through a review of medical records and advertisements and underwent a comprehensive screening process that included a graded treadmill exercise test to assess cardiovascular fitness, with the full testing protocol described in the Physical Function subsection.

Eligibility criteria included men aged ≥65 years with obesity (BMI ≥30 kg/m²), confirmed hypogonadism (fasting morning total testosterone levels <10.4 nmol/L on two occasions), mild-to-moderate frailty (PPT score 18-31) (25), sedentary lifestyle (<1 hr/week of regular exercise), and stable weight and medications for ≥ 6 months. Exclusion criteria included severe cardiopulmonary disease, musculoskeletal limitations precluding exercise, recent use of bone-active drugs, prostate cancer or venous thromboembolism, untreated sleep apnea, hematocrit >50%, and abnormal prostate exam/PSA. After meeting eligibility criteria, participants were block-randomized by BMI (<35 or ≥35 kg/m²) to ensure balance between groups and allocated to LT+TRT or LT+Pbo, with interventions as follows:

LT comprised a structured diet and supervised exercise. The diet aimed for a daily caloric deficit of 500–750 kcal, with weekly group sessions for adjustments, behavioral counseling, and reviews of food diaries. Exercise, held three times per week for 90 minutes, combined aerobic, resistance, and balance training. Aerobic training (e.g., treadmill walking, cycling, stair climbing) began at ~65% of peak heart rate and progressed to 75–85%. Resistance training targeted upper- and lower-body muscles, using weight-lifting equipment, with 1–2 sets of 8–12 reps at 65% of 1-repetition maximum (1-RM) and advancing to 2–3 sets at ~85% of 1RM. The overall goal was a ~10% weight loss in 6 months.

TRT consisted of transdermal testosterone gel (Androgel 1.62%, AbbVie) applied topically once daily in the morning. The initial dose (40.5 mg/day) was selected based on standard dosing guidelines and titrated to achieve serum testosterone concentrations within the physiological range. Testosterone levels were measured 2 weeks after treatment initiation, and the dose was adjusted by an unblinded physician if needed, with repeat testing after another 2 weeks. To maintain blinding, LT+Pbo participants underwent identical procedures and simultaneous sham dose adjustments.

Additional details about the interventions, including compliance data, have been previously described (23).

### Physical function and body composition

Frailty was assessed using the PPT, a composite of nine functional tasks (e.g., stair climbing, walking, chair stands, lifting), each scored 0–4, with a total possible score of 36 (25). Muscle strength was measured by 1-RM testing (biceps curls, bench press, seated row, knee extension, knee flexion, leg press). Peak oxygen consumption (VO_2_peak) was assessed during graded treadmill walking using a Parvo Medics TrueOne 2400 metabolic measurement system. Participants completed a 3–5-minute warm-up at 0% grade, after which treadmill speed was adjusted to each individual’s fastest comfortable walking space. Speed was held constant during the test, while the grade was increased by 2–3% every 2 minutes until the participants became too fatigued to continue. Cardiorespiratory data were collected at 30-second intervals using a computerized metabolic system. This graded treadmill protocol has been described and utilized in our prior work (26).

Body composition and BMD were measured by dual-energy X-ray absorptiometry (DXA; Hologic, Marlborough, MA, USA) (23, 26). Total fat mass and lean body mass, as well as areal BMD of the total hip and lumbar spine were assessed using standard manufacturer protocols and analyzed with APEX Software (version 5.5.2). Thigh muscle volume was assessed by MRI (Siemens Magnetom Avanto, Analyze Directo Software 10.0) using our previously validated protocol (23), which provides reproducible, site-specific quantification of contractile tissue. All imaging procedures were conducted by certified technicians using established quality-control protocols.

### Hormones and metabolic function

Morning fasting blood samples were collected at baseline and after 6 months of intervention. Serum total was measured using a validated liquid chromatography–tandem mass spectrometry LC-MS/MS) assay at Mayo Clinic Laboratories. Free testosterone was measured by equilibrium dialysis, also performed at Mayo Clinic laboratories. Cardiometabolic risk factors were assessed using components of the metabolic syndrome, including fasting glucose, triglycerides, HDL cholesterol, systolic and diastolic blood pressure, and waist circumference. Fasting glucose, triglycerides, and HDL cholesterol were measured using automated assays on a Beckman Coulter DxC800 analyzer in the hospital clinical laboratory. Blood pressure was measured in duplicate after a 5-minute seated rest using a calibrated sphygmomanometer, and waist circumference was measured in triplicate at the midpoint between the lowest rib and the iliac crest. A metabolic syndrome score was calculated from these five components, as previously described, with higher scores indicating greater metabolic dysfunction (24).

### Muscle biopsies

Muscle biopsies were obtained from the vastus lateralis using Tilley-Henkel forceps. Lidocaine (2%) was administered at the biopsy site, followed by a small incision (~1 cm) to allow insertion of the forceps into the vastus lateralis to obtain skeletal muscle tissue. Immediately after collection, specimens were cleaned of blood and adipose tissue, snap-frozen in liquid nitrogen, and stored for batched metabolomic analyses. End-of-intervention biopsies were performed in the morning after an overnight fast and at least 48–72 hours after the last supervised exercise session to minimize acute exercise effects.

### Metabolomics analysis

Muscle metabolomic analyses were performed at the Baylor College of Medicine Metabolomics Core as previously described (27). The targeted panel included metabolites from glycolysis, the PPP, carnitine metabolism, and the TCA cycle as previously described (28–30). Approximately 20 mg of frozen skeletal muscle was homogenized in methanol containing an equimolar mixture of isotopically labeled internal standards to ensure normalization across samples. Homogenates were subjected to biphasic solvent extraction (water/methanol/chloroform, 1:4:3). The aqueous phase containing polar metabolites was isolated, filtered using 3 kDa molecular weight cutoff filters, and dried under vacuum. All participant samples were processed as individual biological samples and were extracted and analyzed together within the same analytical batch to minimize inter-assay variability. A separate pooled QC sample, prepared from small aliquots of representative extracts, was injected periodically throughout the run to monitor instrument stability and performance.

Mass spectrometry analysis was performed using liquid chromatography–triple quadrupole mass spectrometry (LC-QQQ) operated in Single Reaction Monitoring (SRM) mode. Transitions were optimized for each metabolite class, including glycolysis (28), carnitine metabolism (29), the TCA cycle (30), and the PPP. Instrument parameters included a capillary voltage of 3000 V, source temperature of 350 °C, drying gas flow of 10 L/min, nebulizer pressure of 35 psi, and collision energy ranging from 10 to 40 eV, depending on the metabolite.

To ensure reproducibility, isotopically labeled internal standards were added to all samples, and pooled QC muscle extracts were injected periodically to monitor instrument performance. Blank injections were run between samples to prevent carryover. All reagents and consumables were obtained from the same lot to minimize batch effects (27). The complete list of LC–MS reagents and catalog numbers is now provided in **Supplementary Table S1**.

### Statistical analyses

Baseline characteristics were compared between groups using independent t-tests or Fisher’s exact tests. Longitudinal changes in hormones, body composition, physical and metabolic function, and metabolite concentrations were analyzed using repeated measures ANCOVA, with baseline values entered as covariates (SAS Software version 9.4). For metabolite concentrations, data were log-transformed before analysis to meet assumptions of normality and homoscedasticity. Change scores were calculated as the difference between log_2_-transformed values at 6 months and baseline, equivalent to the log_2_ fold change (6 mo/baseline). Between-group differences in change were the primary focus; within-group changes from baseline were also estimated from the same models and are reported in the tables.

Principal component analysis (PCA) was employed to reduce dimensionality and summarize the variation in metabolites within each metabolic domain. For each metabolic pathway, the factor score represented the standardized first principal component derived from metabolites with loadings ≥0.5 (i.e., the “glycolysis factor score” reflected the coordinated variation among glycolytic intermediates). Change scores for PCA-derived factor scores (glycolysis, PPP, carnitines, and TCA cycle) were calculated as the difference between 6-month and baseline values, adjusted for baseline, and then standardized. A single component was retained for each domain, making rotation unnecessary. Component loadings ≥0.5 were used to generate standardized component scores. PCA analyses were conducted separately for each metabolite class to maintain domain specificity. The primary analysis for metabolomics focused on the between-group difference in change in PCA-derived factor scores, which were pre-specified secondary outcomes of the trial. No adjustment for multiple comparisons was applied. Exploratory analyses of individual metabolites were also conducted to illustrate patterns within each pathway, and these within-group comparisons were considered hypothesis-generating.

To evaluate relationships between metabolic domains and clinical outcomes, Pearson’s correlation coefficients (r) were used to assess associations between changes in PCA-derived factor scores and changes in functional, metabolic, and body composition/BMD outcomes. Results are reported as mean ± SE, and all statistical tests were two-sided, with *p* < 0.05 considered statistically significant.

## Results

### Intervention effects on clinical, functional, and metabolic outcomes

Baseline characteristics, including demographics and BMI, were well-balanced between groups. As expected, TRT produced larger increases in total and free testosterone than placebo, consistent with treatment assignment and as summarized in **Table 1**. Despite comparable weight loss in both groups, LT+TRT preserved muscle mass and hip BMD to a greater extent, indicating that TRT helped attenuate the catabolic effects of weight loss. Physical function outcomes improved overal**l,** with VO_2_peak increasing more with TRT, suggesting a modest additive effect on aerobic capacity, whereas other measures of physical function (PPT, gait speed, and 1-RM strength) improved similarly in both groups. Triglyceride levels declined and the metabolic syndrome score improved with LT in both groups, reflecting favorable metabolic effects of the intervention overall.

**Table 1:** Baseline characteristics and key effects of lifestyle therapy ± TRT on hormones, body composition, bone mineral density, physical function, and metabolic function.

Variable	LT+TRT (*n* = 22)	LT+Pbo (*n* = 22)	Difference (95% CI)	Between group *p* value*
Age (yr.)	73.1 ± 0.7	72.9 ± 0.7		0.82
Race/ethnicity, n (%)				0.95
White	9 (41)	10 (45)		
Black	10 (45)	9 (41)		
Hispanic	3 (14)	3 (14)		
Body mass index (kg/m^2^)	36.6 ± 1.2	35.7 ± 0.9		0.54
Male hormones				
Total testosterone (nmol/l)				
Baseline	8.6 ± 0.6	8.5 ± 0.5		
Change at 6 months	11.7 ± 0.9†	4.2 ± 0.9†	-7.4 (-11 to -3.9)	<0.001
Free testosterone (nmol/l)				
Baseline	0.21 ± 0.02	0.22 ± 01		
Change at 6 months	0.44 ± 0.05†	0.08 ± 0.05†	-0.35 (-0.53 to -0.17)	<0.001
Body composition				
Body weight (kg)				
Baseline	112.7 ± 4.5	112.4 ± 3.1		
Change at 6 months	-9.7 ± 0.7†	-10.9 ± 0.7†	-1.2 (-4.5 to 2.0)	0.45
Thigh muscle volume (cm^3^)				
Baseline	1646.3 ± 39.5	1616.6 ± 55.4		
Change at 6 months	-21.0 ± 10.9	-71.2 ± 10.9†	-48.7 (-93.5 to -4.0)	0.03
Bone mineral density				
Total hip (gm/cm^2^)				
Baseline	1.109 ± 0.041	1.123 ± 0.030		
Change at 6 months	0.008 ± 0.003§	-0.015 ± 0.003‡	-0.023 (-0.334 to -0.024)	<0.001
Physical function				
VO_2_peak (mL/kg/min)				
Baseline	17.6 ± 1.9	18.0 ± 0.6		
Change at 6 months	4.2 ± 0.4†	2.5 ± 0.4†	-1.7 (-3.2 to -0.0)	0.04
Metabolic function				
Triglyceride (mmol/l)				
Baseline	1.4 ± 0.1	1.5 ± 0.1		
Change at 6 months	-0.4 ± 0.1†	-0.4 ± 0.1†	-0.1 (-0.4 to 0.1)	0.47
Metabolic syndrome score¶				
Baseline	4.7 ± 0.3	5.3 ± 0.4		
Change at 6 months	-2.7 ± 0.2†	-2.9 ± 0.2†	-0.3 (-1.2 to 0.5)	0.43

Values are mean ± SE unless otherwise indicated. Baseline values are observed means, and change values are least-squares means derived from mixed-model repeated-measures ANCOVA, adjusted for baseline.

*p values represent between-group comparisons of change from baseline.

†*p* < 0.001 for the comparison of within-group change from baseline.

‡*p* < 0.01 for the comparison of within-group change from baseline.

§*p* < 0.05 for the comparison of within-group change from baseline

¶Metabolic syndrome score was calculated from fasting glucose, HDL cholesterol, triglycerides, waist circumference, and mean blood pressure, as described previously (24).

Clinical outcomes in this subgroup are consistent with those in the parent trial and are shown for context (23).

LT+TRT, lifestyle therapy (diet and exercise training) plus TRT; LT+Pbo, lifestyle therapy plus placebo; VO_2_peak, peak oxygen consumption; 1-RM, one-repetition maximum.

To illustrate individual variability, participant-level change scores for all main outcomes in **Table 1** are shown in **Supplementary Figure 1**. Key clinical, functional, and metabolic outcomes are summarized in **Table 1**, while additional measures—including extended body-composition variables, lumbar-spine BMD, and individual metabolic-syndrome components—are presented in **Supplementary Table S2**.

### Differential metabolite changes between LT+TRT and LT+Pbo across key pathways

**Figure 2:**
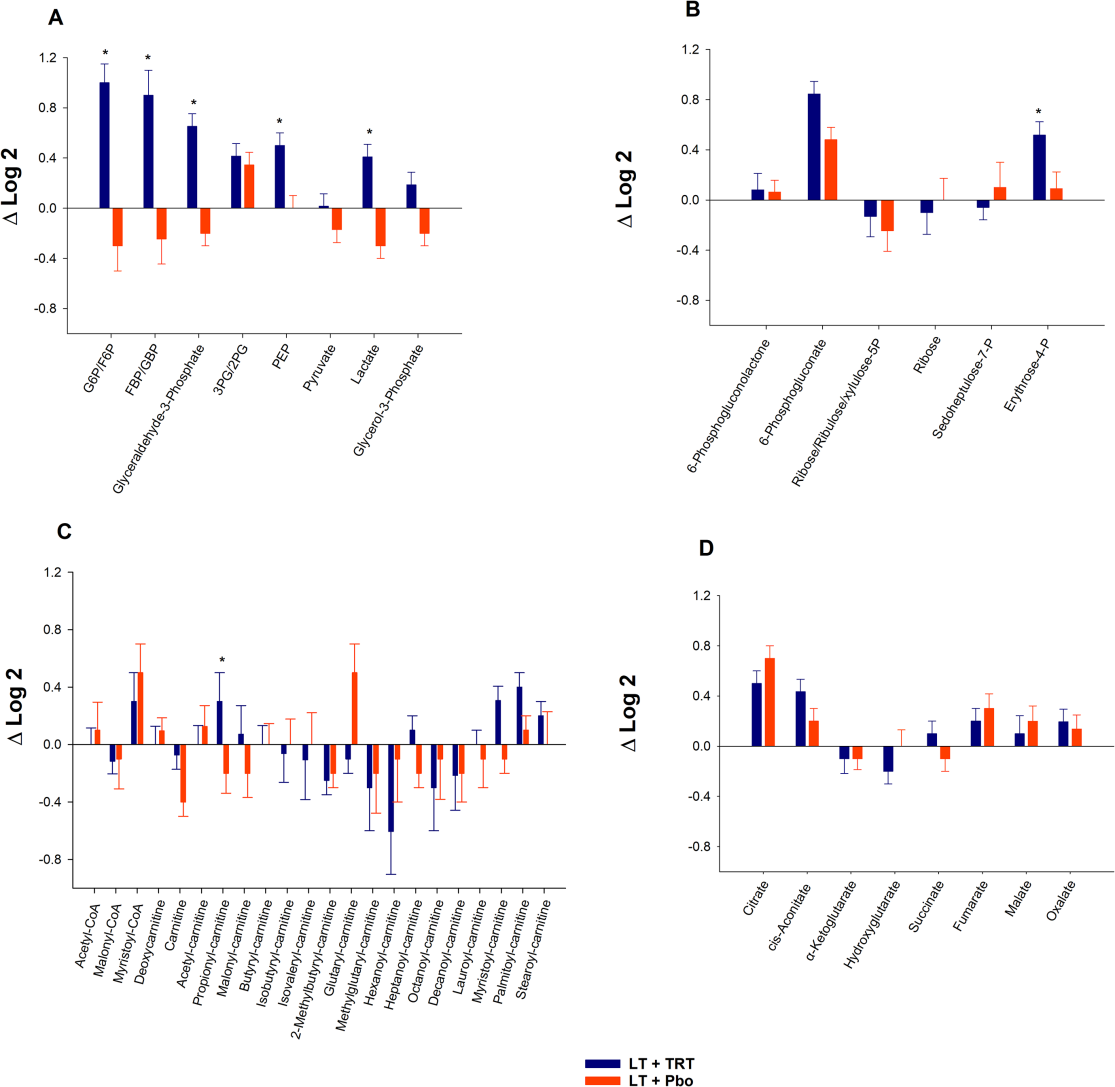
Differential metabolite changes between LT+TRT (lifestyle therapy + testosterone replacement therapy) and LT+Pbo (lifestyle therapy + placebo) across key pathways. **(A)** Glycolysis. **(B)** Pentose phosphate pathway. **(C)** Carnitine metabolism. **(D)** Tricarboxylic acid cycle. Bars represent mean ± SE of Δlog_2_ (6-months − baseline) in metabolite abundance. *P < 0.05, significantly different compared with LT+Pbo. Metabolite abbreviations are defined in **Supplementary Table S2**.

We first examined individual metabolites across the major metabolic pathways. In glycolysis, LT+TRT was associated with greater increases in G6P/F6P, FBP/GBP, glyceraldehyde-3-phosphate, PEP, and lactate compared with LT+Pbo, whereas 3PG/2PG, pyruvate, and glycerol-3-phosphate showed no differences (**Figure 2A**; **Supplementary Table S3**). In the PPP, erythrose-4P was significantly higher in LT+TRT than in LT+Pbo, while other metabolites were unchanged (**Figure 2B**; **Supplementary Table S3**). In carnitine metabolism, propionyl-carnitine was significantly elevated in LT+TRT, with no between-group differences for other acylcarnitines (**Figure 2C**; **Supplementary Table S3**). In the TCA cycle, no significant metabolite differences were detected (**Figure 2D**; **Supplementary Table S3**). **Supplementary Table S3** also provides baseline values and within-group changes.

### Principal component analysis

To reduce dimensionality and capture coordinated variation within each metabolic pathway, we performed PCA. The first component in glycolysis, pentose, carnitine, and TCA metabolism had eigenvalues of 3.87, 2.49, 7.69, and 2.74, explaining 55.2%, 41.5%, 35.0%, and 34.3% of the variance, respectively (**Table 2**). In glycolysis, the component was most strongly represented by glyceraldehyde-3-phosphate (0.95) and lactate (0.93). The pentose component was driven primarily by 6-Phosphogluconate (0.88) and erythrose-4P (0.84). Carnitine metabolism was characterized by butyryl-carnitine (0.91), octanoyl-carnitine (0.90), and isobutyryl-carnitine (0.90). The TCA cycle component was dominated by cis-aconitate (0.84) and malate (0.73), with smaller contributions from fumarate (0.69) and citrate (0.68).

**Table 2:** Principal component loadings, eigenvalues, and cumulative variance for metabolic pathways.

Constituents	Loadings	Eigenvalue	Cumulative variance
**Glycolysis**		3.87	55.2%
Glyceraldehyde 3-phosphate	0.95		
Lactate	0.93		
FBP/GBP	0.88		
G6P/F6P	0.86		
3PG and 2PG	0.59		
PEP	0.35		
Pyruvate	-0.37		
**Pentose**		2.49	41.5%
6-Phosphogluconolactone	0.88		
Erythrose-4P	0.84		
Ribose-Ribulose-Xylulose-5P	0.71		
Ribose	0.71		
Sedoheptulose-7P	0.52		
6-Phosphogluconate	0.47		
**Carnitine metabolism**		7.69	35.0%
Butyryl-carnitine	0.91		
Octonyl-carnitine	0.90		
Isobutyryl-carnitine	0.90		
Heptanoyl-carnitine	0.89		
Methylglutaryl-carnitine	0.89		
Decanoyl-carnitine	0.88		
Hexanoyl-carnitine	0.84		
Lauroyl-carnitine	0.82		
Myristoyl-carnitines	0.60		
Glutaryl-carnitine	0.53		
Isovaleryl-carnitine	0.48		
2-Methylbutyryl-carnitine	0.43		
Palmitoyl-carnitine	0.39		
**Tricarboxylic acid cycle**		2.74	34.3%
Cis-Aconitate	0.84		
Malate	0.73		
Fumarate	0.69		
Citrate	0.68		
Hydroxyglutarate	0.56		
α-Ketoglutarate	0.37		

Factor loadings ≥0.50 were considered to contribute substantially to the component. Eigenvalues represent the amount of variance explained by each component; cumulative variance indicates total variance accounted for.

FBP = fructose-1,6-bisphosphate; GBP = glucose-1,6-bisphosphate; G6P = glucose-6-phosphate; F6P = fructose-6-phosphate; 3PG = 3-phosphoglycerate; 2PG = 2-phosphoglycerate; PEP = phosphoenolpyruvate

### LT+TRT increases glycolysis factor score compared with LT+Pbo

**Figure 3:**
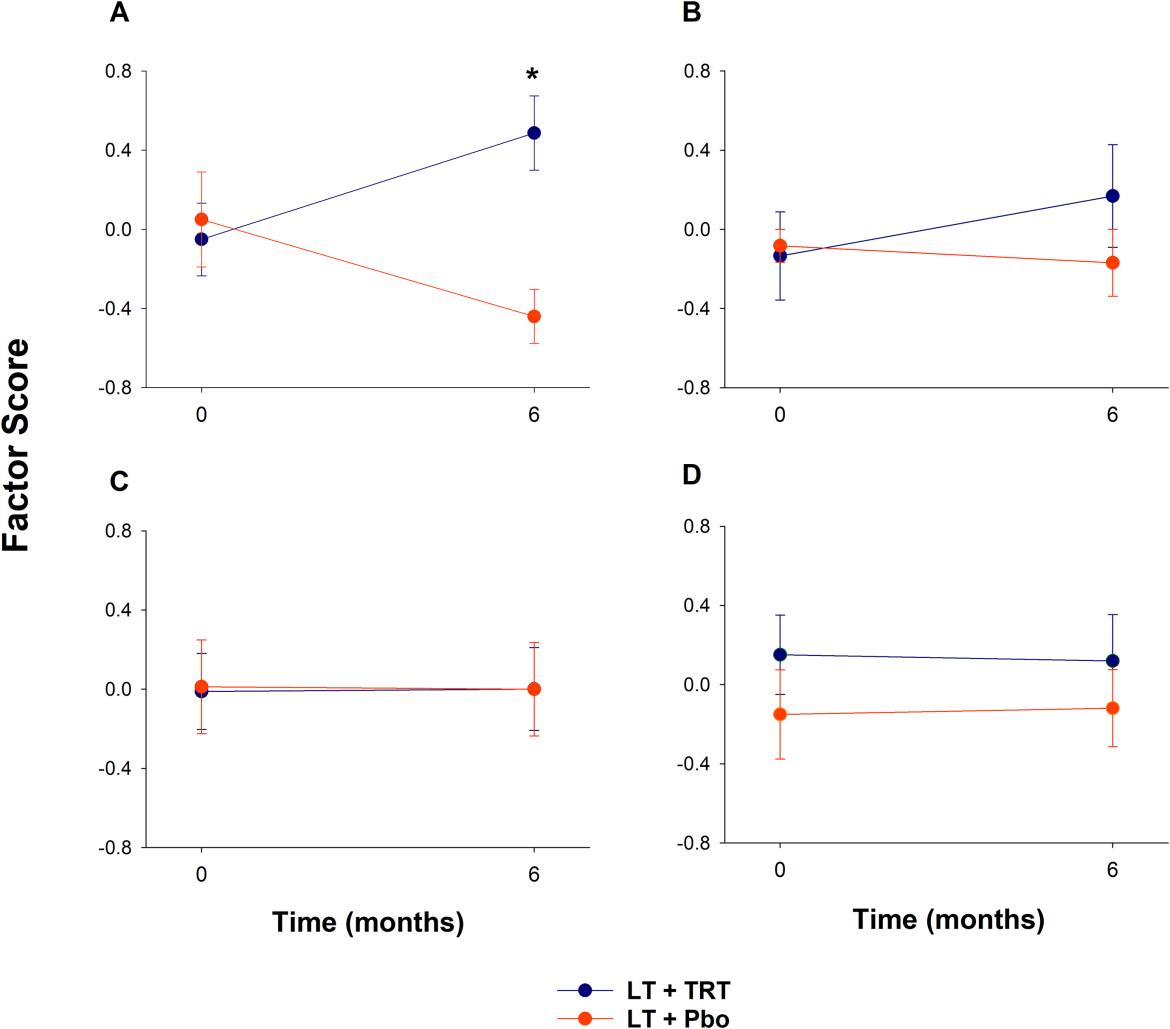
Longitudinal changes in principal component factor scores for metabolic pathways in LT+TRT (lifestyle therapy + testosterone replacement therapy) and LT+Pbo (lifestyle therapy + placebo). **(A)** Glycolysis. **(B)** Pentose phosphate pathway. **(C)** Carnitine metabolism. **(D)** Tricarboxylic acid cycle. Values represent mean ± SE of factor scores at baseline and 6 months. *P < 0.05, significantly different compared with LT+Pbo.

Using the PCA-derived factor scores, which were pre-specified secondary outcomes, we evaluated longitudinal changes in metabolic pathways (**Figure 3**). In glycolysis, the factor score increased significantly in LT+TRT compared with LT+Pbo (*p* = 0.005). The two groups demonstrated opposite trajectories: glycolytic activity increased in LT+TRT but declined in LT+Pbo (**Figure 3A**). In the PPP, a non-significant upward trend was observed in LT+TRT, whereas scores in LT+Pbo remained unchanged (**Figure 3B**). In carnitine metabolism (**Figure 3C**) and the TCA cycle (**Figure 3D**), factor scores remained stable in both groups, with no significant between-group differences.

### Associations between glycolysis and functional and metabolic outcomes

**Figure 4:**
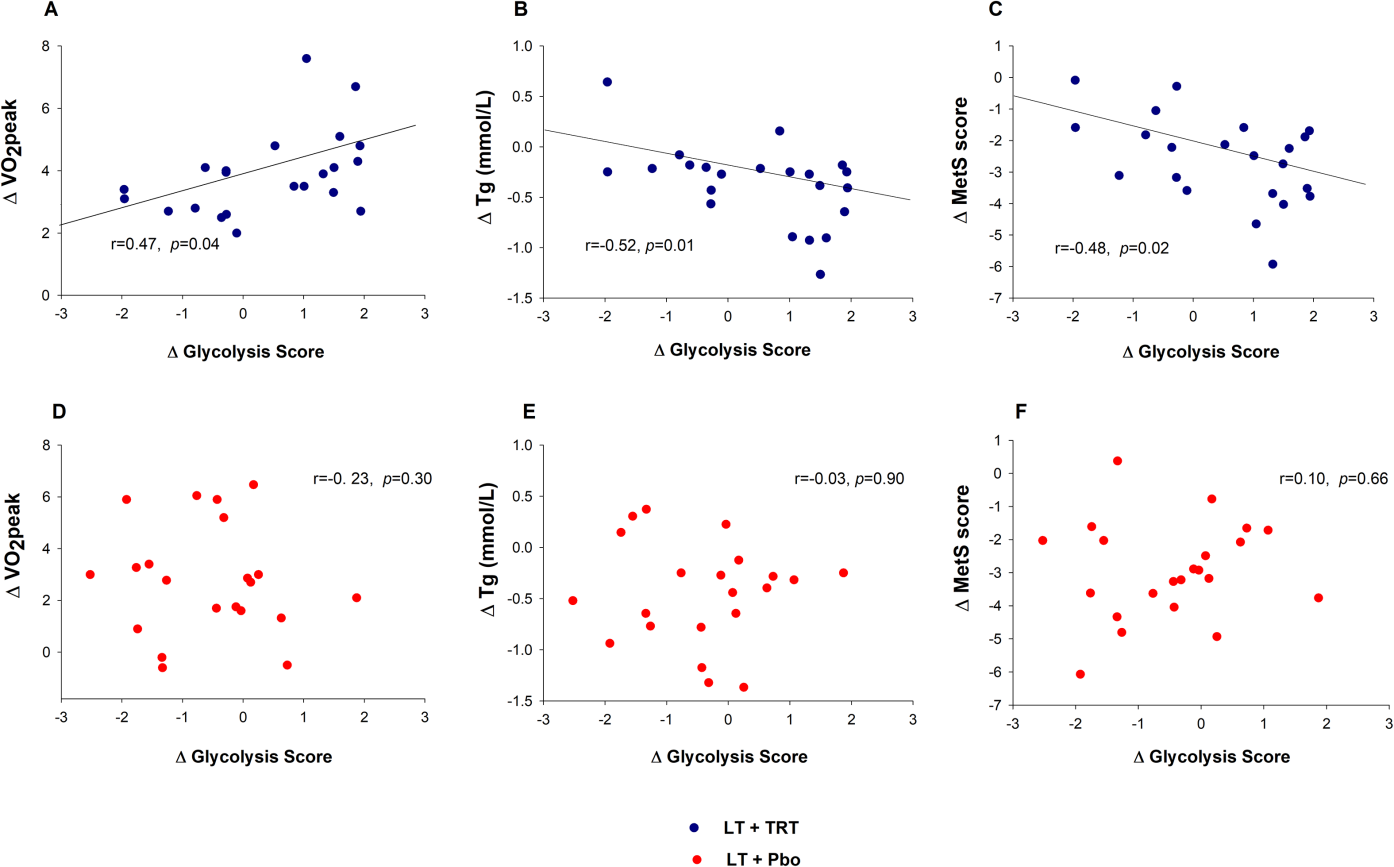
Associations between changes in glycolysis factor score and clinical outcomes. **(A–C)** Higher Δ glycolysis factor score correlated with improved Δ peak oxygen consumption (VO_2_peak, mL/kg/min), lower triglycerides, and reduced metabolic syndrome score (MetS) in LT+TRT (lifestyle therapy + testosterone replacement therapy). **(D–F)** No significant associations were observed in LT+Pbo (lifestyle therapy + placebo). Pearson’s r and *P* values are shown.

Correlation analyses linked changes in glycolysis factor scores to functional and metabolic outcomes (**Figure 4**). In the LT+TRT group, an increase in the glycolysis factor score was positively correlated with improvements in VO_2_peak (r=0.47, *p* = 0.04; **Figure 4A**). In contrast, glycolysis factor score changes were inversely correlated with triglyceride levels (r=−0.52, *p* = 0.01; **Figure 4B**) and with metabolic syndrome score (r=−0.48, *p* = 0.02; **Figure 4C**). No significant correlations were observed with muscle mass, muscle strength, or BMD. In the LT+Pbo group, no significant associations were detected. (**Figures 4D–F**).

**Figure 5:**
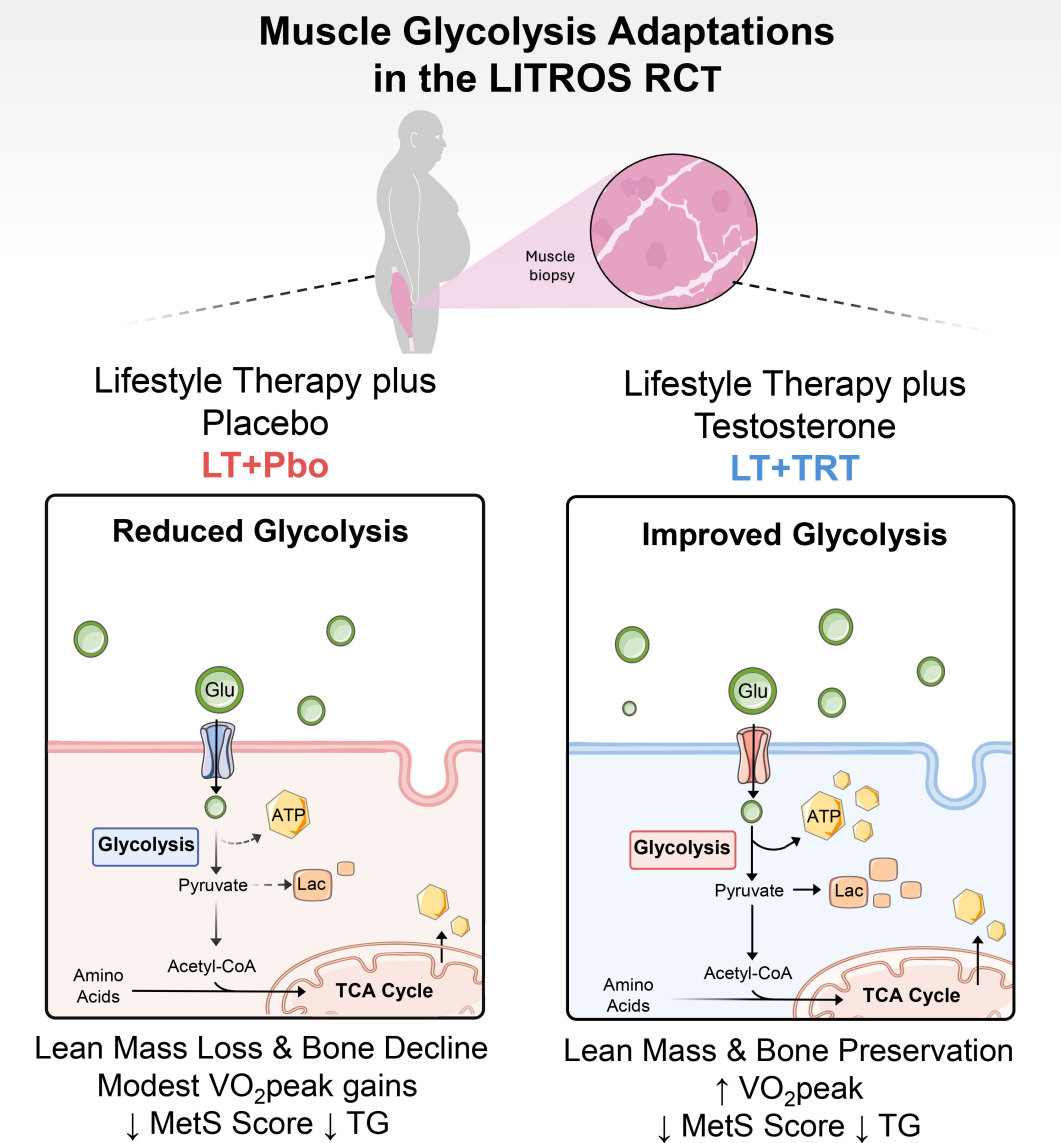
Schematic summary of glycolysis-related metabolic patterns during lifestyle therapy plus testosterone replacement (LT+TRT) and lifestyle therapy plus placebo (LT+Pbo). Lifestyle therapy plus testosterone (LT+TRT) showed a pattern consistent with improved glycolysis, accompanied by preservation of lean mass and bone, greater increases in VO_2_peak, and reductions in metabolic syndrome score and triglycerides. Lifestyle therapy plus placebo (LT+Pbo) was characterized by reduced glycolysis, lean mass loss, modest VO_2_peak gains, and smaller improvements in metabolic risk factors. The schematic illustrates group differences observed in the metabolomic substudy.

A schematic summary of glycolysis-related metabolic patterns distinguishing LT+TRT from LT+Pbo is shown in **Figure 5**.

## Discussion

The LITROS muscle metabolomic substudy was designed to explore the metabolic mechanisms underlying our previous LITROS trial, which demonstrated that TRT combined with LT mitigates the loss of muscle and bone mass during weight loss in older men with obesity and hypogonadism (23). Although several studies have investigated the individual effects of CR (31–33), physical activity (PA) (34–36), or anabolic androgenic steroids (15, 16, 37) on the metabolome, this is, to our knowledge, the first study to examine the combined impact of CR, PA, and TRT on the skeletal muscle metabolomic profile of frail older adults with obesity and hypogonadism. Among the pathways examined, glycolysis emerged as the dominant and consistent metabolic signature of LT+TRT, highlighting a unique molecular adaptation that was not observed with LT+Pbo.

This observation is notable because glycolysis represents a fundamental energy-producing pathway that not only generates ATP rapidly but also supplies key intermediates to biosynthetic and oxidative processes (38, 39). Because the glycolysis factor score in our study is derived from circulating metabolite concentrations rather than gene expression, it reflects coordinated changes in glycolysis-related metabolites. In older men with obesity and hypogonadism, metabolic flexibility is impaired, reflected by a reduced capacity to switch between fatty acid and glucose oxidation in response to energetic demands (15, 40, 41). Our results suggest that TRT restores this capacity in part by improving glycolysis, thereby counteracting the decline observed in the placebo. In the LT+Pbo group, glycolysis factor scores decreased, indicating a shift toward greater oxidative reliance. While this may reflect improved oxidative efficiency, it also represents a loss of glycolytic capacity, which may compromise the ability to generate rapid ATP and anabolic intermediates during energetic stress. By contrast, TRT increased glycolysis, providing it the necessary energetic and anabolic support to maintain lean tissue, including both muscle and bone mass, as well as functional capacity during weight loss.

Mechanistically, this LT+TRT-induced metabolic reprogramming was characterized by coordinated elevations of glycolytic intermediates in both the preparatory (energy-investment) and payoff phases (energy-producing), which we interpreted as consistent with enhanced glycolytic flux and a shift toward the reduction of pyruvate to lactate. This metabolic pattern provides ATP under conditions of energetic demand and may contribute to improved exercise performance. Importantly, glycolysis intermediates such as glucose-6-phosphate and 3-phosphoglycerate can also feed ancillary anabolic pathways, including the PPP and serine biosynthesis (42, 43). Although these pathways did not show coherent long-term reprogramming in our dataset, their potential to be engaged acutely underscores the multifaceted role of improved glycolysis in supporting resilience.

Clinically, these molecular adaptations were linked to meaningful outcomes. Within LT+TRT, higher increases in the glycolysis factor score correlated with greater improvements in VO_2_peak, reductions in triglyceride levels, and lower metabolic syndrome scores, whereas no such associations were observed in LT+Pbo. These findings are consistent with prior evidence that TRT may enhance aerobic capacity and cardiometabolic health, and extend this literature by providing direct muscle-level evidence of improved glycolytic reprogramming (42, 44). A plausible biological explanation is the lactate shuttle; enhanced glycolysis increases lactate availability, which is then oxidized by mitochondria in adjacent fibers or other tissues, thereby coupling anaerobic glycolysis to aerobic ATP production (45). This process supports oxidative metabolism during exercise because lactate produced through glycolysis can serve as a readily oxidizable substrate for mitochondrial respiration, allowing individuals with greater glycolytic activation to sustain higher aerobic work rates. In parallel, androgen-mediated improvements in GLUT4 expression and trafficking (46, 47) may augment muscle glucose uptake and systemic insulin sensitivity, contributing to reductions in triglycerides and overall metabolic risk (48). Beyond muscle, preserved glycolytic support may also contribute to the maintenance of bone mass, consistent with our prior phenotypic finding (22, 23), where combined interventions preserved skeletal integrity during weight loss.

Importantly, these metabolomic results may also be consistent with our prior transcriptomic findings in the same cohort (22). In that substudy, LT+TRT attenuated the downregulation of skeletal muscle metabolic pathways observed with LT+Pbo, potentially including genes linked to glucose utilization and glycolysis. While detailed alignment at the gene level remains to be confirmed, this pattern suggests a degree of multi-omics coherence, with both transcriptomic and metabolomic data indicating the preservation of muscle glucose metabolism under TRT. Future studies should therefore integrate transcriptomic, metabolomic, and proteomic analyses to establish how anabolic interventions remodel metabolic networks in aging muscle.

Our findings extend prior observations on the metabolic consequences of hypogonadism. Plasma metabolomics studies in hypogonadal men have shown suppressed glycolytic flux, enhanced amino acid–derived gluconeogenesis, and greater reliance on ketogenesis (17). The accumulation of lactate and acetyl-CoA, diverted toward ketone body production, reflects a state of metabolic inflexibility that may impair both muscle function and systemic glucose handling. By contrast, our present results provide direct evidence that TRT can improve this trajectory at the muscle tissue level.

The novelty of this study lies in combining a mechanistic metabolomic approach with a carefully designed lifestyle intervention trial in frail older men with obesity and hypogonadism. Unlike previous studies on CR or exercise training alone, which mainly promote oxidative adaptations (32–36) or studies of TRT monotherapy, focused primarily on body composition and strength (11, 12, 42, 44), our findings show a unique synergy—TRT specifically enhanced glycolysis in the context of combined diet and exercise. This change was accompanied by the preservation of lean mass and BMD and was directly associated (through observed correlations) with improvements in aerobic capacity and cardiometabolic health. Together, these data build on earlier clinical findings from LITROS (23) and offer a new mechanistic rationale for understanding how anabolic interventions interact with lifestyle therapy.

A key strength of this work is the robust design of the parent trial, which was randomized and double-blind, minimizing bias and enhancing validity. The supervised and standardized LT program ensured adherence across both groups. At the same time, the targeted muscle metabolomics approach provided a physiologically relevant window into tissue-level remodeling, rather than relying on circulating markers alone. This tissue-based approach is particularly valuable because it captures direct effects within skeletal muscle—the primary effector of both exercise adaptation and glucose disposal—that might be obscured when measuring systemic metabolites.

Despite these strengths, several limitations must be acknowledged. First, the sample size of the metabolomics substudy was modest, which may limit statistical power and generalizability; however, the consistent molecular and clinical patterns observed across multiple endpoints help mitigate this concern. In addition, because we did not apply formal FDR correction, individual metabolite results should be interpreted cautiously within the context of our predefined, pathway-focused hypotheses. Second, the follow-up duration was only six months, which limits our ability to determine the durability of a metabolic shift in this population. Third, ethical considerations prevented inclusion of a TRT-only arm, which limits our ability to determine whether the observed effects reflect TRT alone or its interaction with lifestyle therapy. Fourth, although coordinated changes across glycolytic and related intermediates provide strong inferential evidence of altered pathway activity, metabolite abundances do not directly quantify enzymatic flux, and isotopic or enzymatic validation was not performed. Fifth, while targeted metabolomics provided precision in evaluating glycolysis, PPP, TCA, and carnitine pathways, it also excluded other potentially relevant metabolites. Finally, adherence to TRT remains a real-world challenge, with discontinuation rates of up to 60–70% within one year in clinical practice (49). Although these limitations temper generalizability, the consistency of our findings across molecular, phenotypic, and functional outcomes strengthens confidence in their validity.

These metabolic adaptations, together with observed improvements in aerobic capacity and metabolic risk factors, may have potential implications for managing metabolic and functional decline in older hypogonadal men. Future studies should extend these findings by incorporating complementary omics approaches to deepen understanding of metabolic regulation under testosterone replacement and lifestyle therapy. Larger and longer-term clinical trials are needed to determine the durability and clinical relevance of these metabolic adaptations, including their effects on physical function, disability reduction, and cardiometabolic risk. In addition, study designs that include a testosterone-only arm may help clarify the independent metabolic contributions of testosterone.

## Conclusion

In summary, adding TRT to LT in older men with obesity and hypogonadism improved glycolysis, the only metabolic pathway consistently reprogrammed at six months. This glycolytic adaptation was associated with gains in VO_2_peak, reductions in triglycerides, and lower metabolic syndrome scores, coinciding with the preservation of lean and bone mass. Together, these findings underscore both mechanistic and clinical relevance, suggesting that TRT may help restore metabolic flexibility and resilience in this high-risk population. Larger, longer-term, and mechanistically integrated studies will be essential before translating these results into clinical practice.

## Data Availability

Data generated in this study have been deposited in the NIH database of Genotypes and Phenotypes (dbGaP) under controlled access, in accordance with NIH Genomic Data Sharing Policy and institutional requirements. The dbGaP accession number for this study is phs004472.v1.p1.

## Supplementary material

Supplementary Figure 1 Individual participant change scores (mean ± SE) for key clinical and functional outcomes, including total testosterone, free testosterone, body weight, thigh muscle mass, total hip BMD, VO_2_peak, triglycerides, and metabolic-syndrome score. Each dot represents one participant; box overlays represent group means ± SE for visual reference. LT+TRT = lifestyle therapy plus testosterone; LT+Pbo = lifestyle therapy plus placebo.
