# Supplementary material for: Testosterone plus lifestyle therapy improves skeletal muscle glycolysis in older men with obesity and hypogonadism
Source: Front Endocrinol (Lausanne). 2026 Feb 9;16:1719749. doi: 10.3389/fendo.2025.1719749 (PMC12914099; doi:10.3389/fendo.2025.1719749)
Supplement: Supplementary Figure 1 — Individual participant change scores (mean ± SE) for key clinical and functional outcomes, including total testosterone, free testosterone, body weight, thigh muscle mass, total hip BMD, VO2peak, triglycerides, and metabolic-syndrome score. Each dot represents one participant; box overlays represent group means ± SE for visual reference. LT+TRT = lifestyle therapy plus testosterone; LT+Pbo = lifestyle therapy plus placebo. [file Image1.pdf]

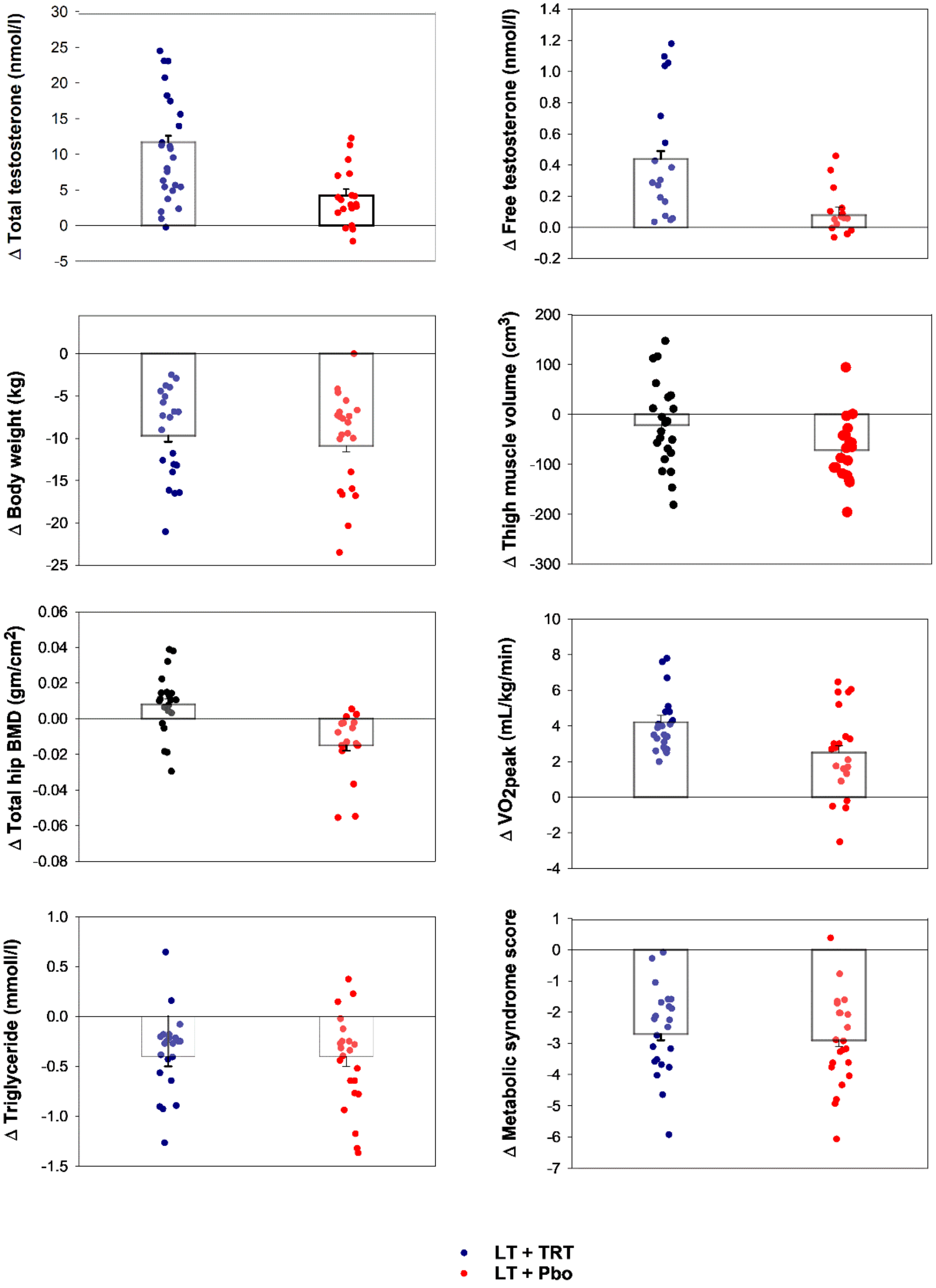
Δ Total testosterone (nmol/l)
Δ Free testosterone (nmol/l)
Δ Body weight (kg)
Δ Thigh muscle volume (cm³)
Δ Total hip BMD (gm/cm²)
Δ VO2peak (mL/kg/min)
Δ Triglyceride (mmoll/l)
Δ Metabolic syndrome score
LT + TRT
LT + Pbo

Supplementary Figure 1. Individual participant change scores (mean ± SE) for key clinical and functional outcomes, including total testosterone, free testosterone, body weight, thigh muscle mass, total hip BMD, VO_2peak, triglycerides, and metabolic-syndrome score. Each dot represents one participant; box overlays represent group means ± SE for visual reference. LT+TRT = lifestyle therapy plus testosterone; LT+Pbo = lifestyle therapy plus placebo.
