# Supplementary material for: Testosterone plus lifestyle therapy improves skeletal muscle glycolysis in older men with obesity and hypogonadism
Source: Front Endocrinol (Lausanne). 2026 Feb 9;16:1719749. doi: 10.3389/fendo.2025.1719749 (PMC12914099; doi:10.3389/fendo.2025.1719749)
Supplement: Supplementary file 2 [file Table1.docx]

**Table S1. Reagents and Consumables Used in the LC-MS Metabolomics Workflow**

| **Reagent** | **Vendor** | **Catalog #** |
| --- | --- | --- |
| LC-MS grade methanol | Fisher Scientific | Cat# A456 |
| LC-MS grade water | Fisher Scientific | Cat# W64 |
| LC-MS grade acetonitrile | Fisher Scientific | Cat# A9554 |
| LC-MS grade formic acid | Sigma-Aldrich | Cat# 5.43804.0250 |
| Ammonium acetate | Sigma-Aldrich | Cat# A1542 |
| Chloroform | Sigma-Aldrich | Cat# 650498 |
| Calibration Solution | Agilent Technologies | Cat# G1969-85000 |
| Gibberellic acid | Sigma-Aldrich | Cat# G7645 |
| Trans-Zeatin | Sigma-Aldrich | Cat# Z0876 |
| Jasmonic acid | Sigma-Aldrich | Cat# J2500 |
| Anthranilic acid | Sigma-Aldrich | Cat# A89855 |
| Thymine-d4 | Sigma-Aldrich | Cat# 487066 |
| N-acetyl aspartic acid-d3 | Sigma-Aldrich | Cat# 616060 |
| Tryptophan^15^N | Sigma-Aldrich | Cat# 574600 |
| Glutamic acid-d5 | Sigma-Aldrich | Cat# 616281 |

.
