# Supplementary material for: Testosterone plus lifestyle therapy improves skeletal muscle glycolysis in older men with obesity and hypogonadism
Source: Front Endocrinol (Lausanne). 2026 Feb 9;16:1719749. doi: 10.3389/fendo.2025.1719749 (PMC12914099; doi:10.3389/fendo.2025.1719749)
Supplement: Supplementary file 3 [file Table2.docx]

**Table S2. Baseline Characteristics and Expanded Outcomes of Lifestyle Therapy ± TRT on Hormones, Body Composition, Bone Mineral Density, Physical Function, and Metabolic Function**

|  | **LT+TRT**  **(*n* = 22)** | **LT+Pbo**  **(*n* = 22)** | **Between Group**  ***P* value*** |
| --- | --- | --- | --- |
| Age (yr.) | 73.1 ± 0.7 | 72.9 ± 0.7 | 0.82 |
| Race/ethnicity, n (%) |  |  | 0.95 |
| White | 9 (41) | 10 (45) |  |
| Black | 10 (45) | 9 (41) |  |
| Hispanic | 3 (14) | 3 (14) |  |
| Body mass index (kg/m^2^) | 36.6 ± 1.2 | 35.7 ± 0.9 | 0.54 |
| **Male hormones** |  |  |  |
| Total testosterone (nmol/L) |  |  |  |
| Baseline | 8.6 ± 0.6 | 8.5 ± 0.5 |  |
| Change at 6 months | 11.7 ± 0.9† | 4.2 ± 0.9† | <0.001 |
| Free testosterone (nmol/L) |  |  |  |
| Baseline | 0.21 ± 0.02 | 0.22 ± 01 |  |
| Change at 6 months | 0.44 ± 0.05† | 0.08 ± 0.05† | <0.001 |
| **Body composition** |  |  |  |
| Body weight (kg) |  |  |  |
| Baseline | 112.7 ± 4.5 | 112.4 ± 3.1 |  |
| Change at 6 months | -9.7 ± 0.7† | -10.9 ± 0.7† | 0.45 |
| Lean body mass (kg) |  |  |  |
| Baseline | 66.3 ± 1.7 | 68.2 ± 1.6 |  |
| Change at 6 months | -1.2 ± 0.3† | -2.5 ± 0.3† | 0.04 |
| Thigh muscle mass (cm^3^) |  |  |  |
| Baseline | 1646.3 ± 39.5 | 1616.6 ± 55.4 |  |
| Change at 6 months | -21.0 ± 10.9 | -71.2 ± 10.9† | 0.03 |
| Fat mass (kg) |  |  |  |
| Baseline | 46.4 ± 3.2 | 44.2 ± 1.9 |  |
| Change at 6 months | -8.2 ± 6.1† | -8.8 ± 6.3† | 0.83 |
| **Bone mineral density** |  |  |  |
| Total hip (gm/cm^2^) |  |  |  |
| Baseline | 1.109 ± 0.041 | 1.123 ± 0.030 |  |
| Change at 6 months | 0.008 ± 0.003§ | -0.015 ± 0.003† | <0.001 |
| Lumbar spine (gm/cm^2^) |  |  |  |
| Baseline | 1.306 ± 0.058 | 1.213 ± 0.222 |  |
| Change at 6 months | 0.016 ± 0.006 | 0.003 ± 0.006 | 0.32 |
| **Physical function** |  |  |  |
| VO_2_peak (mL/kg/min) |  |  |  |
| Baseline | 17.6 ± 1.9 | 18.0 ± 0.6 |  |
| Change at 6 months | 4.2 ± 0.4† | 2.5 ± 0.4† | 0.04 |
| Physical Performance Test score |  |  |  |
| Baseline | 28.8 ± 0.6 | 28.7 ± 0.5 |  |
| Change at 6 months | 4.9 ± 0.3† | 5.0 ± 0.3† | 0.81 |
| Gait speed (m/min) |  |  |  |
| Baseline | 79.9 ± 1.9 | 74.8 ± 2.2 |  |
| Change at 6 months | 4.9 ± 0.3§ | 5.0 ± 0.3‡ | 0.81 |
| Total 1-RM strength (kg) |  |  |  |
| Baseline | 314.5 ± 15.0 | 323.2 ± 18.3 |  |
| Change at 6 months | 65.1 ± 6.8† | 71.5 ± 6.6† | 0.63 |
| **Metabolic function** |  |  |  |
| Fasting glucose (mmol/l) |  |  |  |
| Baseline | 6.5 ± 0.4 | 6.9 ± 0.4 |  |
| Change at 6 months | -1.0 ± 3.2‡ | -1.0 ± 0.2‡ | 0.25 |
| HDL Cholesterol (mmol/l) |  |  |  |
| Baseline | 1.2 ± 0.1 | 1.1 ± 0.1 |  |
| Change at 6 months | 0.1 ± 0.0 | 0.2 ± 0.0† | 0.17 |
| Triglyceride (mmol/l) |  |  |  |
| Baseline | 1.4 ± 0.1 | 1.5 ± 0.1 |  |
| Change at 6 months | -0.4 ± 0.1† | -0.4 ± 0.1† | 0.47 |
| Systolic pressure (mm Hg) |  |  |  |
| Baseline | 137.6 ± 2.4 | 133.9 ± 2.4 |  |
| Change at 6 months | -5.4 ± 0.9† | -4.8 ± 0.9§ | 0.54 |
| Diastolic pressure (mm Hg) |  |  |  |
| Baseline | 84.8 ± 1.9 | 13.4 ± 4.8 |  |
| Change at 6 months | -7.1 ± 1.9† | -6.5 ± 1.9‡ | 0.54 |
| Waist circumference (cm) |  |  |  |
| Baseline | 126.9 ± 3.4 | 126.3 ± 2.6 |  |
| Change at 6 months | -9.4 ± 2.2† | -10.3 ± 1.9† | 0.56 |
| Metabolic syndrome score¶ |  |  |  |
| Baseline | 4.7 ± 0.3 | 5.3 ± 0.4 |  |
| Change at 6 months | -2.7 ± 0.2† | -2.9 ± 0.2† | 0.43 |

Values are mean ± SE unless otherwise indicated. Baseline values are observed means, and change values are least-squares means derived from mixed-model repeated-measures ANCOVA, adjusted for baseline.

* *P* values represent between-group comparisons of change from baseline.

† *P*<0.001 for the comparison of within-group change from baseline.

‡ *P*<0.01 for the comparison of within-group change from baseline.

§ *P*<0.05 for the comparison of within-group change from baseline

¶ Metabolic syndrome score was calculated from fasting glucose, HDL cholesterol, triglycerides, waist circumference, and mean blood pressure, as described previously.^24^

Abbreviations: LT+TRT, lifestyle therapy (diet and exercise training) plus TRT; LT+Pbo, lifestyle therapy plus placebo; VO_2_peak, peak oxygen consumption; 1-RM, one-repetition maximum; HDL, high-density lipoprotein.
