# Supplementary material for: Testosterone plus lifestyle therapy improves skeletal muscle glycolysis in older men with obesity and hypogonadism
Source: Front Endocrinol (Lausanne). 2026 Feb 9;16:1719749. doi: 10.3389/fendo.2025.1719749 (PMC12914099; doi:10.3389/fendo.2025.1719749)
Supplement: Supplementary file 4 [file Table3.docx]

**Table S3. Baseline Values and 6-Month Changes in Individual Metabolites (log₂-transformed)**

|  | **LT + TRT**  **(*n* =22)** | **LT + Pbo**  **(*n* = 22)** | **Between Group**  ***P* value*** |
| --- | --- | --- | --- |
| **Glycolysis** |  |  |  |
| G6P/F6P |  |  |  |
| Baseline | 1.8 ± 0.3 | 2.2 ± 0.3 |  |
| Change at 6 months | 1.0 ± 0.2 ‡ | -0.3 ± 0.2 | 0.00 |
| FBP/GBP |  |  |  |
| Baseline | 1.5 ± 0.2 | 1.6 ± 0.2 |  |
| Change at 6 months | 0.9 ± 0.2‡ | -0.2 ± 0.2§ | 0.01 |
| Glyceraldehyde-3-Phosphate |  |  |  |
| Baseline | -2.7 ± 0.1 | -2.6 ± 0.2 |  |
| Change at 6 months | 0.6 ± 0.1§ | -0.2 ± 0.1 | 0.01 |
| 3PG/2PG |  |  |  |
| Baseline | 0.0 ± 0.1 | -0.3 ± 0.2 |  |
| Change at 6 months | 0.5 ± 0.1 ‡ | 0.3 ± 0.1§ | 0.48 |
| Phosphoenolpyruvate |  |  |  |
| Baseline | -3.3 ± 0.1 | -3.1 ± 0.1 |  |
| Change at 6 months | 0.5 ± 0.1† | -0.0 ± 0.1 | 0.02 |
| Pyruvate |  |  |  |
| Baseline | -8.8 ± 0.1 | -8.8 ± 0.1 |  |
| Change at 6 months | 0.0 ± 0.1 | -0.2 ± 0.1 | 0.32 |
| Lactate |  |  |  |
| Baseline | 2.7 ± 0.1 | 2.7 ± 0.2 |  |
| Change at 6 months | 0.4 ± 0.1§ | -0.3 ± 0.1 | 0.01 |
| Glycerol-3-Phosphate |  |  |  |
| Baseline | -0.6 ± 0.1 | -0.5 ± 0.2 |  |
| Change at 6 months | 0.2 ± 0.1 | -0.2 ± 0.1 | 0.13 |
| **Pentose Phosphate Pathway** |  |  |  |
| 6-Phosphogluconolactone |  |  |  |
| Baseline | 1.8 ± 0.3 | 2.2 ± 0.3 |  |
| Change at 6 months | 0.1 ± 0.1 | 0.0 ± 0.1 | 0.92 |
| 6-Phosphogluconate |  |  |  |
| Baseline | -3.6 ± 0.2 | -3.8 ± 0.2 |  |
| Change at 6 months | 0.9 ± 0.1* | 0.5± 0.1§ | 0.14 |
| Ribose/Ribulose/xylulose-5P |  |  |  |
| Baseline | -2.8 ± 0.1 | 2.8 ± 0.1 |  |
| Change at 6 months | -0.1 ± 0.1 | -0.1 ± 0.1 | 0.54 |
| Ribose |  |  |  |
| Baseline | -4.8 ± 02 | -4.8 ± 0.2 |  |
| Change at 6 months | -0.1 ± 0.2 | 0.0 ± 0.2 | 0.83 |
| Sedoheptulose-7-P |  |  |  |
| Baseline | -5.5 ± 0.3 | -5.4 ± 0.2 |  |
| Change at 6 months | -0.1 ± 0.2 | 0.1 ± 0.2 | 0.83 |
| Erythrose-4-P |  |  |  |
| Baseline | -0.1 ± 1.1 | 0.2 ± 0.1 |  |
| Change at 6 months | 0.5 ± 0.1‡ | 0.1 ± 0.1 | 0.03 |
| **Carnitine metabolism** |  |  |  |
| Acetyl-CoA |  |  |  |
| Baseline | -11.9 ± 0.2 | -12.1 ± 0.1 |  |
| Change at 6 months | 0.0 ± 0.1 | 0.1 ± 0.1 | 0.32 |
| Malonyl-CoA |  |  |  |
| Baseline | -12.5 ± 0.2 | -12.8 ± 0.2 |  |
| Change at 6 months | -0.1 ± 0.1 | -0.1 ± 0.1 | 0.91 |
| Myristoyl-CoA |  |  |  |
| Baseline | -1.6 ± 0.2 | -1.4 ± 0.2 |  |
| Change at 6 months | 0.3 ± 0.2 | 0.5 ± 0.2 | 0.60 |
| Deoxy-carnitine |  |  |  |
| Baseline | -0.6 ± 0.1 | -0.7 ± 0.1 |  |
| Change at 6 months | -0.0 ± 0.1 | -0.1 ± 0.1 | 0.47 |
| Carnitine |  |  |  |
| Baseline | 0.9 ± 0.1 | 1.0 ± 0.1 |  |
| Change at 6 months | -0.1 ± 0.1 | -0.4 ± 0.1§ | 0.17 |
| Acetyl-carnitine |  |  |  |
| Baseline | 4.7 ± 0.1 | 4.6 ± 0.1 |  |
| Change at 6 months | 0.0 ± 0.1 | 0.1 ± 0.1 | 0.32 |
| Propionyl-carnitine |  |  |  |
| Baseline | 0.6 ± 0.1 | 0.7 ± 0.1 |  |
| Change at 6 months | 0.3 ± 0.2 | -0.2 ± 0.1 | 0.02 |
| Malonyl-carnitine |  |  |  |
| Baseline | -5.2 ± 0.1 | -5.1 ± 0.1 |  |
| Change at 6 months | 0.1 ± 0.1 | -0.2 ± 0.1 | 0.21 |
| Butyryl-carnitine |  |  |  |
| Baseline | -1.7 ± 0.1 | -1.8 ± 0.2 |  |
| Change at 6 months | 0.0 ± 0.2 | 0.0 ± 0.2 | 0.92 |
| Isobutyryl-carnitine |  |  |  |
| Baseline | -1.7 ± 0.2 | -1.8 ± 0.2 |  |
| Change at 6 months | -0.1 ± 0.2 | -0.0 ± 0.2 | 0.85 |
| Isovaleryl-carnitine |  |  |  |
| Baseline | -0.8 ± 0.3 | -0.8 ± 02 |  |
| Change at 6 months | -0.1 ± 0.2 | -0.3 ± 0.2 | 0.64 |
| 2-Methylbutyryl-carnitine |  |  |  |
| Baseline | -5.2 ± 0.1 | -5.3 ± 0.2 |  |
| Change at 6 months | -0.2 ± 0.1 | -0.2 ± 0.1 | 0.78 |
| Glutaryl-carnitine |  |  |  |
| Baseline | -3.5 ± 0.2 | -3.5± 0.2 |  |
| Change at 6 months | -0.1 ± 0.2 | 0.5 ± 0.2 | 0.32 |
| Methylglutaryl-carnitine |  |  |  |
| Baseline | -7.5 ± 0.3 | -7.3 ± 0.4 |  |
| Change at 6 months | -0.3 ± 0.3 | -0.2 ± 0.3 | 0.99 |
| Hexanoyl-carnitine |  |  |  |
| Baseline | -2.9 ± 0.3 | -3.2 ± 0.4 |  |
| Change at 6 months | -0.6 ± 0.3 | -0.1 ± 0.3 | 0.29 |
| Heptanoyl-carnitine |  |  |  |
| Baseline | 1.6 ± 0.3 | 1.5 ± 0.3 |  |
| Change at 6 months | 0.1 ± 0.1 | -0.2 ± 0.1 | 0.21 |
| Octanoyl-carnitine |  |  |  |
| Baseline | -0.6 ± 0.3 | -0.4 ± 0.3 |  |
| Change at 6 months | -0.3 ± 0.3 | -0.1 ± 0.3 | 0.88 |
| Decanoyl-carnitine |  |  |  |
| Baseline | -0.4 ± 0.3 | -0.4 ± 0.3 |  |
| Change at 6 months | -0.2 ± 0.2 | -0.2 ± 0.2 | 0.95 |
| Lauroyl-carnitine |  |  |  |
| Baseline | -0.3 ± 0.3 | -0.3 ± 0.3 |  |
| Change at 6 months | 0.0 ± 0.2 | -0.1 ± 0.2 | 0.86 |
| Myristoyl-carnitine |  |  |  |
| Baseline | -13.9 ± 0.3 | -14.0 ± 0.2 |  |
| Change at 6 months | 0.3 ± 0.2 | -0.1 ± 0.2 | 0.26 |
| Palmitoyl-carnitine |  |  |  |
| Baseline | -3.5 ± 0.1 | -3.3 ± 0.1 |  |
| Change at 6 months | 0.4 ± 0.1 | 0.1 ± 0.1 | 0.17 |
| Stearoyl-carnitine |  |  |  |
| Baseline | -7.7 ± 0.2 | -7.9 ± 0.2 |  |
| Change at 6 months | 0.2 ± 0.2 | 0.0 ± 0.2 | 0.67 |
| **Tricarboxylic acid cycle** |  |  |  |
| Citrate |  |  |  |
| Baseline | -1.1 ± 0.2 | -1.2 ± 0.2 |  |
| Change at 6 months | 0.5 ± 0.1§ | 0.7 ± 0.1‡ | 0.64 |
| cis-Aconitate |  |  |  |
| Baseline | -12.5 ± 0.2 | -12.8 ± 0.2 |  |
| Change at 6 months | 0.4 ± 0.1 | 0.2 ± 0.1 | 0.18 |
| α-Ketoglutarate |  |  |  |
| Baseline | -0.6 ± 0.1 | -0.7 ± 0.1 |  |
| Change at 6 months | -0.1 ± 0.1 | -0.1 ± 0.1 | 0.62 |
| Hydroxyglutarate |  |  |  |
| Baseline | 0.9 ± 0.1 | 1.0 ± 0.1 |  |
| Change at 6 months | -0.2 ± 0.1 | -0.0 ± 0.1 | 0.39 |
| Succinate |  |  |  |
| Baseline | 4.7 ± 0.1 | 4.6 ± 0.1 |  |
| Change at 6 months | 0.1 ± 0.1 | -0.1 ± 0.1 | 0.44 |
| Fumarate |  |  |  |
| Baseline | -3.0 ± 0.1 | -3.2 ± 0.1 |  |
| Change at 6 months | 0.2 ± 0.1 | 0.3 ± 0.1§ | 0.41 |
| Malate |  |  |  |
| Baseline | 2.6 ± 0.1 | -2.4 ± 0.1 |  |
| Change at 6 months | 0.2 ± 0.1 | 0.1 ± 0.1 | 0.99 |
| Oxalate |  |  |  |
| Baseline | -6.0 ± 0.1 | -6.0 ± 0.1 |  |
| Change at 6 months | 0.2 ± 0.1 | 0.1 ± 0.1 | 0.59 |

Values are mean ± SE unless otherwise indicated. Baseline values are observed means, and change values are least-squares means derived from mixed-model repeated-measures ANCOVA, adjusted for baseline.

* *P* values represent between-group comparisons of change from baseline.

† *P* <0.001 for the comparison of within-group change from baseline.

‡ *P* < 0.01 for the comparison of within-group change from baseline.

§ *P* <0.05 for the comparison of within-group change from baseline

Abbreviations: LT+TRT = lifestyle therapy plus testosterone replacement therapy; LT+Pbo = lifestyle therapy + placebo; G6P = glucose-6-phosphate; F6P = fructose-6-phosphate; FBP = fructose-1,6-bisphosphate; GBP = glucose-1,6-bisphosphate; 3PG = 3-phosphoglycerate; 2PG = 2-phosphoglycerate.
